# 78. Longitudinal Analysis of T cells in COVID-19 Survivors with Post-Acute Sequelae of COVID-19 Reveals Associations between Individual Symptoms and Inflammatory Indexes

**DOI:** 10.1093/ofid/ofac492.003

**Published:** 2022-12-15

**Authors:** Stephanie LaVergne, Stephanie LaVergne, Taru Dutt, Kim McFann, Bridget Baxter, Tracy Webb, Kailey Berry, Sophia Stomberg, Julie Dunn, Marcela Henao-Tamayo, Elizabeth Ryan

**Affiliations:** Kaiser Permanente, Denver, CO; Kaiser Permanente, Denver, CO; Colorado State University, Fort Collins, Colorado; UCHealth, Loveland, Colorado; Colorado State University, Fort Collins, Colorado; Colorado State University, Fort Collins, Colorado; Colorado State University, Fort Collins, Colorado; Colorado State University, Fort Collins, Colorado; UC Health, Fort Collins, Colorado; Colorado State University, Fort Collins, Colorado; Colorado State University, Fort Collins, Colorado

## Abstract

**Background:**

Many individuals infected with SARS-CoV-2 are left with persistent symptoms of COVID-19. Post-acute sequelae of COVID-19 (PASC) can affect quality of life and functionality. The mechanism underlying PASC is unknown but elevated inflammatory markers several months post infection have been found in those with PASC.

**Methods:**

Individuals diagnosed with COVID-19 were evaluated longitudinally for PASC and persistent symptoms. CD4^+^ and CD8^+^ cellular markers and intracellular cytokines were assessed at each follow-up time point and analyzed by individual PASC symptoms reported.

**Results:**

Participants who reported persistent dyspnea, forgetfulness, confusion, and chest pain had significantly higher levels of CD8^+^Ki67^+^ cells. Those with dyspnea also had significantly higher levels of CD8^+^CD38^+^, CD8^+^Granzyme B^+^, and CD8^+^IL10^+^ cells. Those who suffered from forgetfulness, chest pain, and joint pain had significantly higher levels of CD4^+^CD25^+^ cells.

**Conclusion:**

These findings suggest continued CD8^+^ T cell and CD4+CD25^+^ T cell activation and response following SARS-CoV-2 infection in patients with PASC. An increase in T regulatory cells suggests an ongoing attempt to control host inflammation in a subset of these patients. These results shed further light on continued immune system activation and chronic inflammation as a link to symptoms in COVID-19 survivors suffering from PASC.

**Disclosures:**

**All Authors**: No reported disclosures.

**Table 1:**
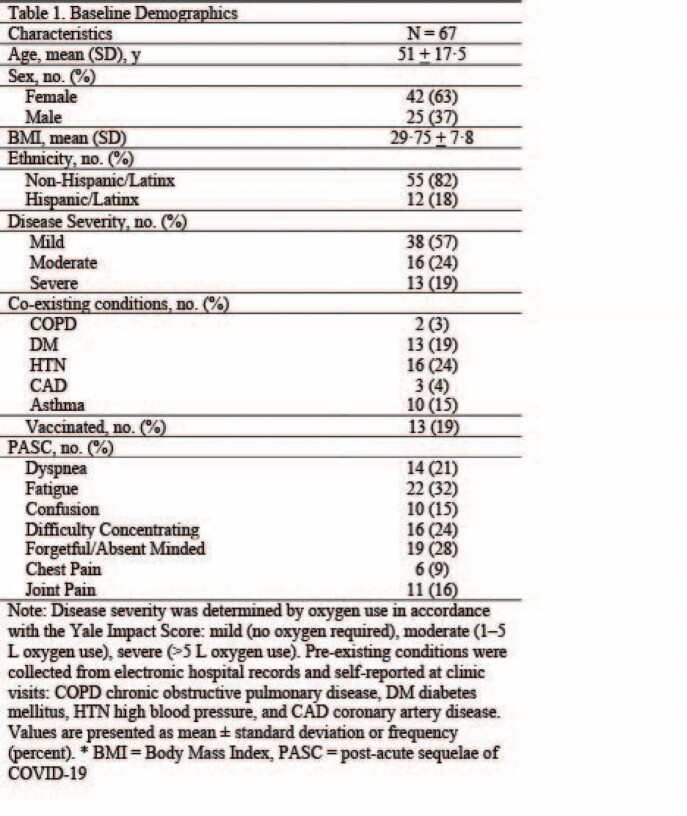
Baseline participant characteristics and demographic data.

**Figure 1:**
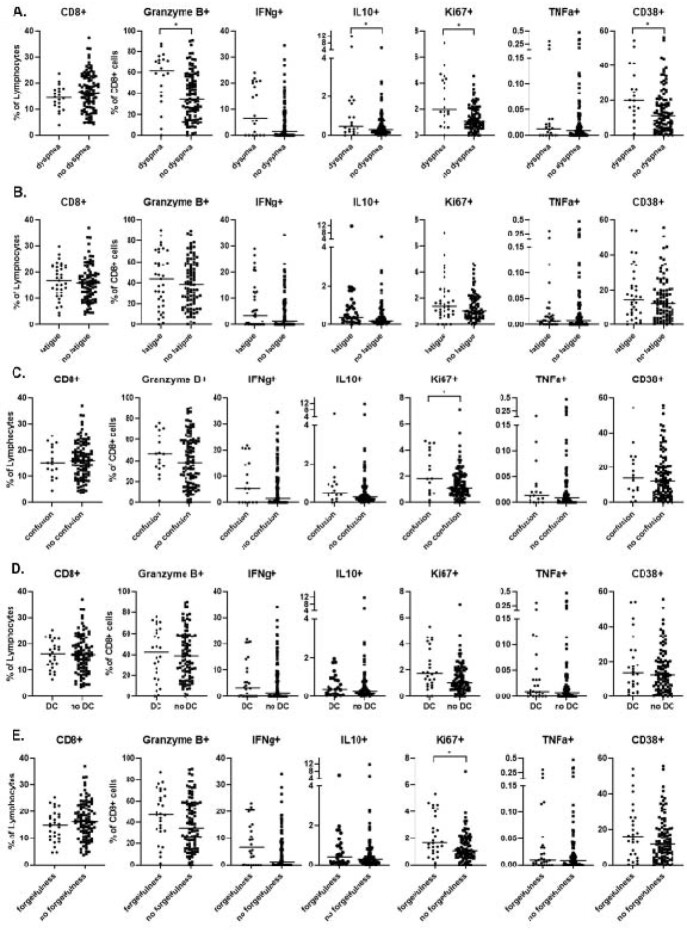
Differences in CD8 T cell markers and inflammatory cytokines in participants reporting symptoms of: a) dyspnea (N=20) and without dyspnea (N=104), b) fatigue (N=35) and without fatigue (N=89), c) confusion (N=17) and without confusion (N=107), d) difficulty concentrating (DC) (N=26) and without difficulty concentrating (N= 98), and e) forgetfulness or absent-mindedness (N=28) and without forgetfulness or absent-mindedness (N=96). *p<0.05.

**Figure 2:**
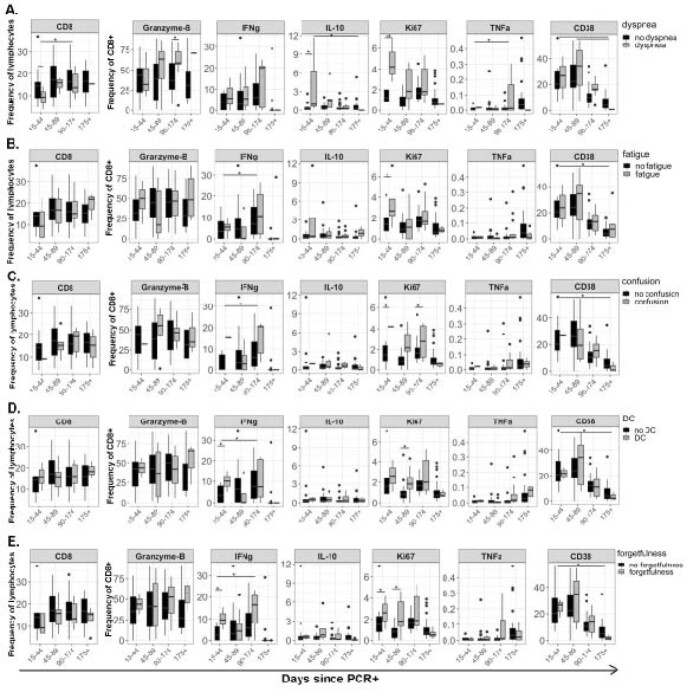
Inflammatory indexes measured at 15–44, 45–89, 90–174, and > 175 days post PCR by symptom: A) dyspnea B) fatigue C) confusion D) DC=difficulty concentrating E) forgetfulness.

